# Progress in Surgery

**Published:** 1897-07-10

**Authors:** 


### Progress in Surgery.

### RHINOLOGY.

(*Continued from p.* 234.)

**Hypertrophic Rhinitis.**—There is a tendency on the part of some rhinologists to refer cases of atrophic rhinitis to too extensive former cauterisation. Bryson Delavan,[4] at the American Laryngological Association, introduced a new method for the permanent relief of certain enlargements of the turbinated bodies. He said that the use of the cautery often produced too great loss of mucous membrane, while affording no permanent relief. Our aim should be to reduce the volume of the congested turbinates. He, therefore, would advocate the method of submucous incisions. For this purpose he used a small knife, first applying cocaine in the usual manner, and then passing the blade under the mucosa, making a sweep through the sub-mucous tissues, and then withdrawing it, care being taken to avoid any additional enlargement of the original opening. The instrument employed was a needle rather than a knife. It was better to repeat the operation than to overdo it. There was but little pain and only slight bleeding. It was a good plan to keep the cocaine in contact with the tissues for several hours, and to let the slight hæmorrhage stop of its own accord. This method of operating possessed the advantages of ease of execution, freedom from bad effects, preservation of the mucosa, and a practical adaptability to the desired end. Perhaps the most serious danger of advanced cases of nasal atrophy and suppuration is aural complications. A large proportion of cases have recurrent attacks of acute eustachian salpingitis, a certain proportion of these going on to suppurative diseases of the middle ear and perforation of the membrana tympani. Especially is this the case where the nasal douche, snuffing fluid from the hand, or similar measures in which hydrostatic pressure is brought to bear, have been employed. Colonies of streptococci are thus easily forced into the eustachian tubes, with the result of setting up suppurative conditions extremely prejudicial to the welfare of the patient.

**Maxillary Sinus.**—The diagnosis of antral empyema is sometimes very difficult despite the many perfected methods of determining the precise character of the antral affection. Generally malignant disease is easily differentiated, but F. S. Milbury,[5] of Brooklyn, reports a case of a man 54 years of age, who was afflicted with a carcinoma in the sinus, which, when first seen, presented much of the physical features encountered in ordinary cases of suppuration of the sinus. There was fœtid mucopurulent secretion, with polypi extending into the pharynx posteriorly, while the whole antral area of the left side was in deep shadow under electric illumination in the mouth. Within 25 days the case terminated fatally. The diagnosis of carcinoma was confirmed by the pathologist of Harvard, after a microscopic examination of a portion of the growth excised for that purpose. Another point of importance is impressed on our minds by Herbert Tilley[6]: "Frontal sinus disease is rare compared to antral, so always treat your case as an antral one until you have proved it otherwise, no matter how intense is the supra-orbital headache, or how earnestly your patient assures you that he is certain his disease is in his forehead. I impress this rule on you because I have now this case under my care, and have reported another[7] where healthy frontal sinuses have been trephined, and now are quite well since the antra have been drained of their pus."

[4] Med. Rec., May 22, 1897. [5] New York Med. Jour., No. 933, 1896. [6] Clin. Jour., April 14, 1897. [7] Lancet, Sept. 26, 1896.

